# The Multifactorial Impact of ApoE4 on Alzheimer's Pathology and Cognitive Decline

**DOI:** 10.1002/alz70855_105101

**Published:** 2025-12-24

**Authors:** Aya Arrar, Madison R Longmuir, Kate M. Onuska, Bryan Lung, Scheila Schmidt, Amr Eed, Kellly Summers, Arash Salahinejad, Ravi S. Menon, Ali R. Khan, Lisa M Saksida, Timothy J Bussey, Tallulah S Andrews, Taylor W Schmitz, Vania F Prado, Marco AM Prado

**Affiliations:** ^1^ Schulich School of Medicine & Dentistry, London, ON, Canada; ^2^ University of Western Ontario, London, ON, Canada; ^3^ Robarts Research Institute, London, ON, Canada; ^4^ Schulich School of Medicine and Dentistry, London, ON, Canada; ^5^ Schulich Medicine and Dentistry, London, ON, Canada; ^6^ Centre for Functional and Metabolic Mapping, Robarts Research Institute, London, ON, Canada; ^7^ Western University, London, ON, Canada; ^8^ Lawson Health Research Institute, London, ON, Canada

## Abstract

**Background:**

Apolipoprotein E (ApoE) isoforms differentially regulate Aβ and tau pathology in Alzheimer's disease (AD). However, most preclinical models do not recapitulate the combined impact of Aβ, tau, and ApoE on cognitive function and disease progression. To overcome this, we developed new mouse models harbouring humanized variants of the ApoE genotypes (ApoE3, ApoE4), hApp (App^WT^, App^NL^ or App^NL‐F^) and hMAPT (tau), and evaluated whether their interactions influence cognition, brain structure, and pathological load.

**Method:**

Methods used include single nuclei RNA sequencing (snRNAseq), magnetic resonance imaging (MRI), immunofluorescence microscopy, ELISAs, and Western Blotting. Attentional function was assessed using the cross‐species Continuous Performance Task, an automated touchscreen test.

**Result:**

Biochemical and immunofluorescence analyses confirm that ApoE genotype interacts with both Aβ and tau to drive distinct pathogenic trajectories. At 6 months, App^NL‐F^/ApoE4 mice exhibit higher cortical levels of insoluble Aβ42 compared to their ApoE3 counterparts, indicating that ApoE4 accelerates amyloidogenic processes. This trend persists at 9 and 12 months, where App^NL‐F^/ApoE4 mice show increased insoluble Aβ42 levels, plaque burden and plaque size compared to App^NL‐F^/ApoE3 mice. Additionally, cortical levels of soluble and insoluble total tau are elevated in App^NL‐F^/ApoE4 mice relative to App^NL‐F^ /ApoE3 mice at 9 and 16 months, respectively. MRI reveals reduced grey matter volume in fronto‐cortical regions of 6‐month‐old App^NL‐F^/ApoE4 mice compared to App^NL^/ApoE3 mice. snRNA‐seq confirms a reduction in cortical excitatory neurons, and an upregulation of gliosis in App^NL‐F^/ApoE4 mice. Finally, App^NL‐F^/ApoE4 mice exhibit attentional deficits on CPT as early as 6 months, with these impairments persisting at 9 and 12 months relative to App^NL‐F^/ApoE3 mice. Pathology was negligible at all timepoints in App^NL^ (ApoE3 and ApoE4) and App^WT^ mice, suggesting that Aβ toxicity drives the pathogenic interactions with ApoE.

**Conclusion:**

These findings highlight the multifactorial impact of ApoE4 in AD, demonstrating that humanized App^NL‐F^/MAPT/ApoE4 mice exhibit biochemical and imaging biomarkers of pathology and a time course of neurodegeneration and cognitive dysfunction that reproduces a human‐like phenotype of late onset AD. This work underscores the value of combining these novel mouse models with translational imaging and cognitive biomarkers for understanding the relationship between pathophysiology and high‐level cognitive function.

